# Clinical Characteristics and Management of Ovarian Vein Thrombosis: A Case Series

**DOI:** 10.3389/fcvm.2022.916920

**Published:** 2022-06-16

**Authors:** Mohammed Alsheef, Yacoub Abuzied, Muteb Alosaimi, Amer Altamimi, Qusai Alwazna, Qusai Almahmood, Noura Ali AlBulushi, Jehan Almutair, Abdul Rehman Zia Zaidi, Jenny Gray, Amani Abu-Shaheen

**Affiliations:** ^1^Internal Medicine and Thrombosis, Medical Specialties Department, King Fahad Medical City, Riyadh, Saudi Arabia; ^2^Spinal Cord Injury Unit, Nursing Department, Rehabilitation Hospital, King Fahad Medical City, Riyadh, Saudi Arabia; ^3^College of Medicine, King Saud University, Riyadh, Saudi Arabia; ^4^Adult Hematology Department, Medical Specialties Department, King Fahad Medical City, Riyadh, Saudi Arabia; ^5^College of Medicine, Al-Imam Muhammad ibn Saudi Islamic University, Riyadh, Saudi Arabia; ^6^College of Medicine, King Saud University, Riyadh, Saudi Arabia; ^7^College of Medicine, AlMaarefa University, Riyadh, Saudi Arabia; ^8^Medical Specialties Department, King Fahad Medical City, Riyadh, Saudi Arabia; ^9^Dentistry Administration, King Fahad Medical City, Riyadh, Saudi Arabia; ^10^Department of Scientific Writing, Research Center, King Fahad Medical City, Riyadh, Saudi Arabia

**Keywords:** ovarian vein thrombosis, postpartum, ovarian vein, pregnancy, OVT

## Abstract

**Background:**

Ovarian vein thrombosis (OVT) is an uncommon condition, occurring in ~1 in every 600–2,000 pregnancies. It is associated with various conditions, including thrombophilia, malignancy, sepsis, intra-abdominal and pelvic inflammatory conditions, pregnancy, and the postpartum period, and specific surgical interventions, particularly gynecological surgeries. Thus, this study aims to identify the associated factors for OVT and elaborate on the standard treatment strategies for its management.

**Methods:**

Retrospective data collection was used. Our study consists of 18 patients diagnosed with OVT between 2005 and 2016; the data was collected from the Health Information Management system at King Fahad Medical City, Riyadh, Saudi Arabia using a standard format.

**Results:**

Our study found that OVT involves the right ovarian vein more often than the left and mainly occurs in women during their postpartum period. These patients other associated factor included hypertension, diabetes, and a higher body mass index (BMI) of above 25 kg/m^2^. The most frequently presenting complaints were abdominal pain and fever. The most common treatment was the administration of enoxaparin (a low molecular weight heparin) for an average duration of one to three months, which resulted in a low recurrence rate of OVT.

**Conclusions:**

Physicians should be vigilant for suspicion of OVT in female patients presenting with lower abdominal pain and fever in their postpartum period. Additionally, it is suggested to use low molecular weight heparin as initial therapy for OVT for one to three months, resulting in a high remission rate.

## Introduction

Ovarian vein thrombosis (OVT) is usually associated with different conditions, including thrombophilia, malignancies, sepsis, abdominal and pelvic inflammatory conditions, pregnancy, and post-surgical (1, 2). It is rare to find idiopathic OVT, but a few cases have previously been reported in the literature, two of those cases were in Saudi Arabia (3, 4).

Clinically, OVT may present nonspecific complaints and clinical findings on physical examination, sometimes mimicking the acute abdomen. Some patients may also present with lower abdominal pain and tenderness, vomiting, fever, and abdominal distension (5–7). In addition, although it is rare, some patients have been reported to have presented with ovarian vein thrombophlebitis (6).

The incidence of OVT during pregnancy has been studied, particularly in the postpartum period, which was found to occur in ~1/600 to 1/2000 pregnancies (8). In pregnancy-related OVTs, it has been found that it usually involves the right ovarian vein (70-−90%) followed by bilateral ovarian vein involvement (11–14%). The most likely explanations for this finding are that the longer right ovarian vein lacks competent valves, the effect of the gravid uterus, and the retrograde flow in the left ovarian vein prevents stasis and ascending infection, thus making the left-sided vein less vulnerable to thrombosis (9).

Like most other venous thromboses, OVT is also associated with various malignancies at different stages; for example, in one published series by Jacoby et al., it was found that gonadal ovarian vein thrombosis is associated with breast and pancreatic cancers. Even though those patients never received any type of anticoagulation, there were no documented complications, and patients remained asymptomatic for the follow-up period, which was almost a year (10). However, there are potentially severe complications in untreated cases, such as pulmonary embolisms and sepsis (10). Given the association of such potentially serious complications, most published reviews suggest using a course of anticoagulation therapy with no agreed-upon recommendations regarding its duration (11, 12).

OVT is often found incidentally on imaging, raising the question of whether to treat such asymptomatic patients or not. Compared to other diagnostic modalities, the computed tomography scan (CT) with contrast has the highest sensitivity and specificity in diagnosing OVT and is widely used as the preferred diagnostic modality for its diagnosis (13). The sensitivity of contrast-enhanced CT to detect OVT approaches 100%, although it may be overlooked by the inexperienced radiologist, given the rarity of the disease (14, 15).

OVT can be managed both medically and surgically, with both strategies having similar success rates (15). However, clinicians are extrapolating from the venous thromboembolism (VTE) guidelines as international guidelines are lacking regarding the management of OVT. Therefore, this study aims to identify the clinical characteristics, associated factors, and management of OVT.

## Materials and Methods

We conducted a case series for all patients diagnosed with OVT since 2005. This was a convenience sample of patients seen in the Thrombosis Clinic at King Fahad Medical City (KFMC), Riyadh, Saudi Arabia, for all cases of OVT seen, their medical record numbers were documented prospectively whenever a case was identified. The inclusion criteria were patients diagnosed with OVT by one of the following clinical tools: Doppler ultrasonography, computed tomography (CT) scan, or magnetic resonance imaging.

The data were collected from the patient's medical record using a standardized case report form.

The following data were collected from all patients: (i) demographic data, which include marital status, age, and weight. high, and body mass index (BMI); (ii) signs and symptoms including vomiting, abdominal pain, abdominal distention, fever, abdominal tenderness, and lower limb swelling; (iii) associated factors and comorbidities including a family history of venous thrombosis, postpartum, malignancy, personal history of VTE, hypertension, diabetes, severe infection or sepsis, inflammatory bowel disease (Ulcerative Colitis or Crohn's Disease), and diverticulitis; (iv) radiological findings which show filling defects in the ovarian vein; (v) lab profile, which included mainly prothrombin time (PT), international normalized ratio (INR), activated partial thromboplastin time (APTT), hemoglobin, platelets count, and creatinine (mean 50.17+12.98 mg/dl); (vi) and treatments used and duration; (vii) recurrence of OVT.

### Ethical Considerations

The study was reviewed and approved by KFMC institutional review board.

### Statistical Analysis

The SPSS version 21 (SPSS Inc., Chicago, Illinois, United States America) was used to enter and analyze data. Descriptive statistics were used in the analysis, which included quantitative data presented as [mean ± standard deviation (SD)] (i.e., age, weight, height, treatment doses, etc...), and qualitative data presented as frequency and percentage (i.e., symptoms and signs of OVT and associated diseases, etc...).

## Results

At the time of the study, 18 patients were diagnosed with OVT. The mean age of the patients was 39.22 ± 10.35 years and the mean BMI was 25.86 ± 6.58 kg/m^2^. OVT was discovered incidentally in one (5.6%) asymptomatic pregnant patient while it was symptomatic in the remaining 17 (94.4%) patients. Abdominal pain was the most common symptom of OVT 15 (83.3%) followed by fever 6 (33.3%), abdominal tenderness 6 (33.3%), and vomiting 3 (16.7%) (Table 1). The most common associated factors for OVT were postpartum 11 (61.1%) followed by malignancy (11.1%), post-laparotomy (5.6%), and idiopathic (5.6%). Seven patients (38.9%) had hypertension, while four patients (22.2%) had diabetes mellitus (Table 1).

**Table 1:** Demographic, clinical characteristics, and associated factors for OVT among the study participants.

**Variables**	***N***	**%**
**Marital status**		
Married	16	89.9%
Unmarried	2	11.1%
Age (years)	39.22 ±10.35 years	
Weight (kg)	63.36 ±15.68 kg	
Height (cm)	155.97 ±4.78 cm	
BMI (kg/m^2^)	
<20	1	5.6%
20–25	6	33.3%
25–30	11	61.1%
**Signs and symptoms**		
Asymptomatic (incidental discovery)	1	5.6%
Vomiting	3	16.7%
Abdominal pain	15	83.3%
Abdominal distention	2	11.1%
Fever	6	33.3%
Abdominal tenderness	6	33.3%
Lower limb swelling	1	5.6%
**Associated factors and comorbidities**		
Postpartum	11	61%
Malignancy	2	11.1%
Personal history of VTE	1	5.6%
Family history of venous thrombosis	0	05
Hypertension	7	38.9%
Diabetes	4	22.2%
Severe infection or sepsis	1	5.6%
Inflammatory bowel disease (Ulcerative Colitis or Crohn's Disease)	1	5.6%
Diverticulitis	1	5.6%

*Data presented either as number and percentage or mean and standard deviation*.

**Figure 1:**
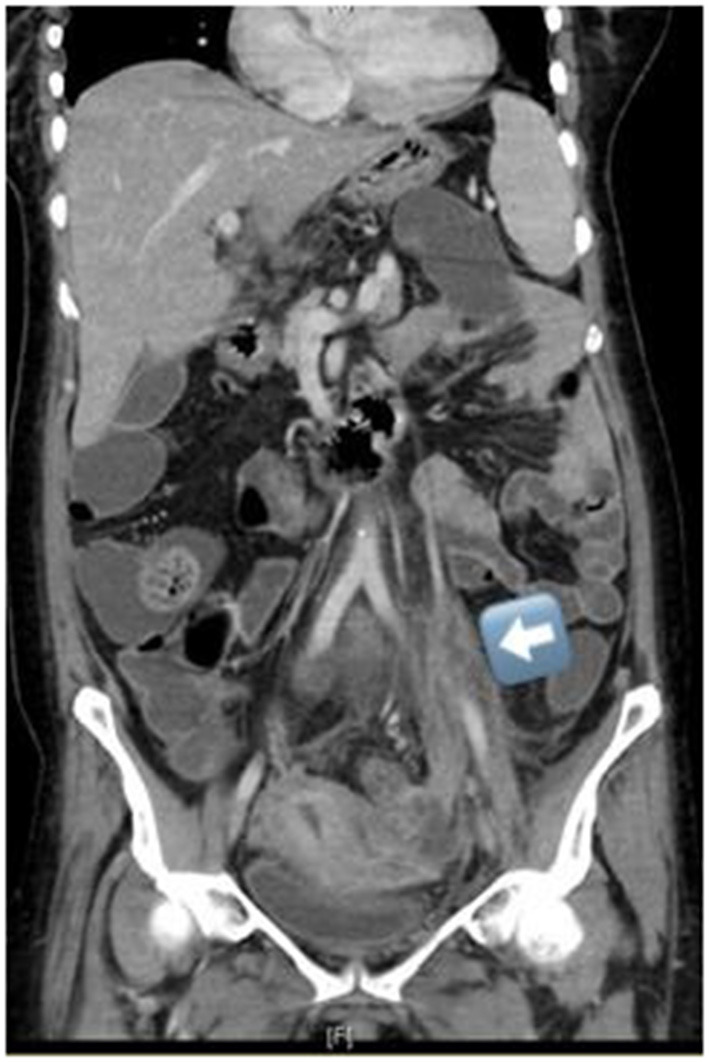
CT abdomen with contrast exhibiting left ovarian vein thrombosis.

CT scans with contrast were used as a diagnostic tool in all of the cases. The most common site of OVT was the right ovarian vein, involving 12 patients (66.7%) followed by the left ovarian vein, five patients (27.8%), while bilateral OVT was found to be in one patient (5.6%) only (Figure 1).

The mean values of different lab investigations were also calculated in our study including PT (mean13.20 ± 1.67 s), INR (mean 1.64 ± 2.41), APTT (mean 35.30 ± 5.80 s), and hemoglobin (mean 10.80 ± 1.98g/dl) (Table 2). The mean INR values were mildly elevated in our sample, which may possibly be explained by elevated Vitamin K levels, however this is not of direct relevance to OVT.

**Table 2:** Lab profile for the study participants.

	**Mean ±SD**	**Normal range**
PT	13.20 ± 1.67sec	9.7–12.6 sec
APTT	35.30 ± 5.80 sec	25.3–38.3 sec
INR	1.64 ±2.41	0.81–1.23
Hemoglobin	10.80 ± 1.98 g/dl	>11.0– <16.0 g/dL
Platelets count	295.11 ± 103.114	100–450 10*3/uL
Creatinine	50.17 ± 12.98mg/dl	49–90 umol

*The underline indicates APTT 35.30 ± 5.80 sec 25.3–38.3 sec activated partial thromboplastin time*.

Enoxaparin was the initial therapy in all patients, but it was given for different durations. The duration of this treatment was 1-3 months in 13 (72.2%) patients, 3–6 months in one (5.6%) patient, six months-1 year in two (11.1%) patients, and lifelong in two (11.1%) patients (Table 3). The optimal duration of anticoagulation for the treatment of OVT remains uncertain. The mean dose of enoxaparin was 59 ± 23.06 mg taken twice daily in nine (60%) patients and once daily in six (40%) patients. The recurrence of OVT was found only in one (5.6%) patient.

**Table 3:** Treatment and duration for managing OVT.

	***N***	**%**
**Treatment**		
Enoxaparin	18	100
**Duration of the treatment**
1–3 months	13	72.2
3–6 months	1	5.6
6 months-−1 year	2	11.1
Extended	2	11.1

## Discussion

Our case series is one of largest case series published in the literature. Previously, four case reports on ovarian vein thrombosis were published in Saudi Arabia, two in the postpartum period, and two were idiopathic (16–20). Many prior studies (1, 8) established that OVT is most commonly associated with the postpartum period, consistent with our findings. The association of OVT with a postpartum period is related to the hypercoagulability status due to the surge of estrogen during pregnancy and the compression of the gravid uterus on the ovarian vein. Our study showed two cases with acute gastrointestinal inflammatory conditions, including diverticulitis and ulcerative colitis, also consistent with previous literature. The medical conditions were not found to be relevant, anemia can be presumed to be due to the underlying medical conditions rather than a precursor to OVT. D-dimer tests were not ordered for the patients as it would not have been reliable considering that the majority of patients were postpartum.

In our study, two cases were associated with malignancy, one secondary to ovarian cancer and the other carcinoma of the cervix. The most common cancer associated with OVT in the literature is ovarian cancer. There was only one case with herpes simplex virus (HSV) infection with herpetic encephalitis, which raised the question of herpes being a potential associated factor. However, none of the previous studies support this. In addition, our study did not find any relation between OVT and the family history of VTE, even in a single case. In this study, we had two patients who had malignant masses before developing OVT of the left ovarian vein, suggesting an association between malignancy and site of OVT (10, 21). In addition, our study had 11 patients with diabetes and hypertension, however, we did not find any relationship between OVT and diabetes and hypertension to augment this association in the literature and this might be explained by the high rates of these conditions in the Saudi population.

In addition, the mean age in our study is 40, and most cases are postpartum; it might be explained by multiparity and delivery during later years which is fairly common in Saudi Arabia. Our patients' two most common symptoms were abdominal pain and fever, consistent with the previous studies (7). One of our patients was misdiagnosed with acute appendicitis, similar to a previous case published in Saudi Arabia.

Our study found that right OVT is the most commonly involved, in line with other findings in the literature. Various anatomic and physiologic factors predispose the right ovarian vein to thrombosis. The increased incidence during pregnancy may be explained by the dextrorotation of the uterus leading to compression of the inferior vena cava and right ovarian vein. Antegrade blood flow and the presence of multiple incompetent valves in the right ovarian vein are other factors favoring bacterial infection compared to retrograde blood flow in the left ovarian vein (22). Pregnancy poses an additional risk due to the ovarian vessels' increased diameter due to hormonal changes and increased blood flow. These changes substantially increase pressure on both the vessel walls and the venous valves resulting in venous incompetence, compounding venous stasis (23). Additionally, the right ovarian vein entry into the inferior vena cava (IVC) at an acute angle makes it more susceptible to compression than the left vein typically, which enters the left renal vein at a right angle (24, 25). The fact that the right ovarian is more commonly involved is consistent with our study with 12 patients (66.7%) involving the right-sided vein.

According to some studies, the sensitivity and specificity of an abdominopelvic CT scan with intravenous contrast is almost 100% and should therefore be considered the initial investigative step. In addition, it is more cost-effective and readily obtainable than magnetic resonance imaging (MRI) (26).

OVT can be managed both medically and surgically, with both strategies having similar success rates. The main medical management strategies include anticoagulants and a seven to 10 days' course of broad-spectrum antibiotics. While the role of surgery in the initial management of OVT is controversial, it is preferred by some clinicians for complicated cases only, such as recurrent pulmonary emboli despite medical treatment, free-floating thrombosis, and contraindication to anticoagulant use (22). Unfortunately, we do not have data about antibiotic use in these series. Recurrence of pregnancy-related OVT is low in subsequent pregnancies. However, it is recommended to give anticoagulant prophylaxis to patients with an underlying hypercoagulable state during pregnancy and the postpartum period. In our study, enoxaparin was the initiation therapy in all patients, 13 (72.2%) of whom received it for 1–3 months with only one patient having a recurrence of OVT, suggesting that this duration is an excellent treatment strategy for OVT.

The optimal duration of anticoagulation for the treatment of OVT remains uncertain. Moreover, international guidelines are lacking regarding the management of OVT. However, we are extrapolating from the venous thromboembolism (VTE) guidelines. The duration of anticoagulation depends on whether the VTE is provoked or unprovoked. The American Society of Hematology (ASH) recommendation for anticoagulation duration for provoked VTE is three months and extended for unprovoked VTE. The American College of Clinical Pharmacy (ACCP) and ASH suggest using direct oral anticoagulants (DOAC) over warfarin to treat VTE in the childbearing period; however, the DOAC Summary of product characteristics are not recommended using DOAC for pregnant and lactating women.

## Limitations

The retrospective design is the main limitation of this study, where potential confounding of other unmeasured factors could affect the results and potentially missing data from the patients' charts. Secondly, data were collected from a single-center, limiting the sample size. Lastly, there was no control group to compare outcomes and assess risk. Hence, a multi-center case-control study is needed. Finally, a detail regarding the use of antibiotics was lacking.

## Conclusion

Physicians should be vigilant and be prepared to suspect OVT in female patients presenting in their postpartum period with lower abdominal pain and fever. Additionally, it is suggested to use low molecular weight heparin as initial therapy for patients with an OVT for 1–3 months' post-diagnosis as this results in a high remission rate.

## Data Availability Statement

The original contributions presented in the study are included in the article/supplementary material; further inquiries can be directed to the corresponding authors.

## Ethics Statement

The studies involving human participants were reviewed and approved by King Fahad Medical City—IRB. Written informed consent for participation was not required for this study in accordance with the national legislation and the institutional requirements.

## Author Contributions

All authors listed have made a substantial, direct, and intellectual contribution to the work and approved it for publication.

## Conflict of Interest

The authors declare that the research was conducted in the absence of any commercial or financial relationships that could be construed as a potential conflict of interest.

## Publisher's Note

All claims expressed in this article are solely those of the authors and do not necessarily represent those of their affiliated organizations, or those of the publisher, the editors and the reviewers. Any product that may be evaluated in this article, or claim that may be made by its manufacturer, is not guaranteed or endorsed by the publisher.
